# Identification of altered miRNAs and their targets in placenta accreta

**DOI:** 10.3389/fendo.2023.1021640

**Published:** 2023-03-03

**Authors:** José M. Murrieta-Coxca, Emanuel Barth, Paulina Fuentes-Zacarias, Ruby N. Gutiérrez-Samudio, Tanja Groten, Alexandra Gellhaus, Angela Köninger, Manja Marz, Udo R. Markert, Diana M. Morales-Prieto

**Affiliations:** ^1^ Department of Obstetrics, Placenta Lab, Jena University Hospital, Jena, Germany; ^2^ Friedrich Schiller University Jena, Faculty of Mathematics and Computer Science, RNA Bioinformatics and High Throughput Analysis, Jena, Germany; ^3^ Faculty of Mathematics and Computer Science, Bioinformatics Core Facility, Friedrich Schiller University Jena, Jena, Germany; ^4^ Department of Gynecology and Obstetrics, University of Duisburg-Essen, Essen, Germany; ^5^ University Department of Gynecology and Obstetrics, Hospital St. Hedwig of the Order of St. John, University Medical Center Regensburg, Regensburg, Germany; ^6^ Fritz Lipman Institute (FLI), Leibniz Institute for Age Research, Jena, Germany

**Keywords:** pregnancy, placenta accreta spectrum, microRNA, RNA-sequencing, miRNA targets, placenta, trophoblast, mRNA

## Abstract

Placenta accreta spectrum (PAS) is one of the major causes of maternal morbidity and mortality worldwide with increasing incidence. PAS refers to a group of pathological conditions ranging from the abnormal attachment of the placenta to the uterus wall to its perforation and, in extreme cases, invasion into surrounding organs. Among them, placenta accreta is characterized by a direct adhesion of the villi to the myometrium without invasion and remains the most common diagnosis of PAS. Here, we identify the potential regulatory miRNA and target networks contributing to placenta accreta development. Using small RNA-Seq followed by RT-PCR confirmation, altered miRNA expression, including that of members of placenta-specific miRNA clusters (e.g., C19MC and C14MC), was identified in placenta accreta samples compared to normal placental tissues. *In situ* hybridization (ISH) revealed expression of altered miRNAs mostly in trophoblast but also in endothelial cells and this profile was similar among all evaluated degrees of PAS. Kyoto encyclopedia of genes and genomes (KEGG) analyses showed enriched pathways dysregulated in PAS associated with cell cycle regulation, inflammation, and invasion. mRNAs of genes associated with cell cycle and inflammation were downregulated in PAS. At the protein level, NF-κB was upregulated while PTEN was downregulated in placenta accreta tissue. The identified miRNAs and their targets are associated with signaling pathways relevant to controlling trophoblast function. Therefore, this study provides miRNA:mRNA associations that could be useful for understanding PAS onset and progression.

## 1 Introduction

Placenta Accreta Spectrum (PAS) is the term that integrates the different grades of abnormal placental adherence and invasion (1, 2). The most current classification of PAS includes three grades: 1. Placenta accreta or adherenta, 2. Placenta increta and 3. Placenta percreta. In adherent placenta accreta, the decidua basalis is partly or completely lost so that the trophoblast layer is directly apposed to the myometrial tissue, but without invading it. Placenta increta is defined by deep invasion of trophoblast cells into the myometrium, and placenta percreta when trophoblast cells invade and penetrate the uterine serosa (2, 3). An additional classification recently suggested by FIGO included the subclassification of grade 3a: Placenta percreta limited to the uterine serosa grade 3b: Placenta percreta with urinary bladder invasion; and grade 3c: Placenta percreta with the involvement of pelvic organs (2–4). PAS severity can be evaluated during pregnancy by ultrasound examinations (5, 6), but many cases remain undiagnosed antepartum and are later classified based on the intraoperative situation and histological findings related to partial or complete loss of decidua basalis and the depth of myometrial trophoblast cell invasion (2, 3, 5). The most common diagnosis of PAS cases is placenta accreta (>70%), followed by increta (~15%) and percreta (10%) (5, 7, 8).

The differences in the clinical and histopathological criteria used to define PAS in previous studies make it difficult to compare data and reach a consensus on the etiology of the disease. A failure in normal decidualization caused by previous endometrial damage is currently the most favored hypothesis, but the abnormal invasive capacities of trophoblast cells may also contribute to the disease (9, 10). PAS is frequently associated with a previous cesarean section, which is increasingly applied, thus, the worldwide prevalence of PAS has risen over the last four decades (11, 12). However, only a few investigations have focused on the molecular mechanisms associated with PAS that may also explain its development in women during their first pregnancy. As PAS does not naturally occur in animals and due to its uniqueness to human pregnancy (3, 13), its study in animal models is highly constricted despite one published mouse model (14).

A recent study reported a very low correlation between transcriptome and proteome profiling of PAS samples suggesting a significant role of post-transcriptional regulation (15), which may be mediated by non-coding RNAs including miRNAs. The human placenta harbors miRNAs (20-22 nucleotides size), which regulate its development and functionality (16). This regulation is further demonstrated by the fact that placenta-specific and -associated miRNAs have particular expression patterns during different stages of pregnancy (17–19). As demonstrated in several pioneer studies, dysregulation of specific miRNAs is associated with pregnancy pathologies, including PAS (20–23). Some of the altered miRNAs are linked with intracellular signaling networks implicated in angiogenesis (24), trophoblast apoptosis (20), and epithelial-mesenchymal transition (23).

In this study, next-generation sequencing was used to screen the miRNA signature of PAS placentas and compare it to healthy pregnancies. Altered miRNAs, as well as their targets, were validated and localized in placental tissues. Our results offer molecular elements for understanding the etiology of PAS and promote the identification of markers in PAS.

## 2 Material and methods

The Placenta Lab strictly applies quality management and is certified after DIN EN ISO 9001.

### 2.1 Patient samples

Patients were recruited for the study from the Department of Obstetrics, University Hospital Jena and the Department of Gynecology and Obstetrics, University Hospital Essen, Germany, between 2014 and 2018. The respective ethics committees approved the study according to the Helsinki Declaration on ethical principles for medical research involving human subjects by obtaining consent forms (Amendment to No: 1509-03/05 Jena and 12-5212-BO Essen). Multiple pregnancies, fetal anomalies, and infections were excluded from the study.

Samples obtained in Jena were collected intraoperatively in cases where a postpartum curettage had to be performed due to incomplete placenta or retention of the placenta after delivery, even under administration of uterotonic drugs or controlled cord traction. An additional sample was taken during cesarean section of a patient with placenta praevia, intraoperatively diagnosed to be an abnormal adherent placenta. Placenta tissue samples were taken in all cases from the suspected sight of detachment failure. Histopathological findings such as loss of decidua basalis or the direct trophoblast apposition to the myometrial tissue without invasion were used as confirmers for the diagnosis of placenta accreta. For controls, placental chorionic tissue was collected from normally delivered placentas including (for ISH and IF) or excluding decidua (RNA analysis). Samples were immediately washed with sterile phosphate buffer solution (PBS) and placed in RNA later (cat. No. AM7021; Invitrogen Life Technologies, Darmstadt, Germany) overnight and then stored in cryotubes at -80°C until RNA extraction, or fixed in 4% formalin overnight before paraffin-embedding. A sub-set of 17 samples was used for the initial RNA-Seq analysis and a complete set of 26 samples to perform the validation PCR. Samples for immunostaining and *in situ* hybridization were also selected from the complete set. Clinical characteristics are summarized in **Table 1**.

**Table 1:** Clinical characteristics of PAS samples.

	Initial RNA-Seq			PCR Validation cohort			Immunostaining			ISH		
	NP (n=9)	PAS (n=8)	p- Value	NP (n=14)	PAS (n=12)	p- Value	NP (n=3)	PAS (n=3)	p- Value	NP (n=4)	PAS (n=6)	p- Value
*Maternal age (y)*	28.9 ± 3.0	30.2 ± 7.5	0.62	30.4 ± 3.5	30.6 ± 6.8	0.91	31.3 ± 10.0	38.7 ± 2.9	0.29	30.8 ± 7.2	37.8 ± 2.3	0.05
*Gestational age (w)*	39.8 ± 0.7	39.4 ± 2.2	0.58	39.2 ± 1.5	39.6 ± 1.8	0.51	39.5 ± 1.6	37.9 ± 3.3	0.50	40.1 ± 1.4	36.6 ± 4.7	0.19
*Placenta weight (g)*	641.5 ± 93.2	509.5 ± 45.5	*<0.05	632.8 ± 116.6	536.3 ± 56.9	0.07	664.3 ± 90.7	527.3 ± 112.8	0.18	654.8 ± 70.2	527.3 ± 112.8 ^#^	0.12
*Birth weight (g)*	3512.2 ± 295.2	3164.4 ± 434.4	0.07	3469.3 ± 375.9	3429.2 ± 570.0	0.83	3481.7 ± 123.9	3016.7 ± 767.9	0.36	3598.8 ± 215.6	2814.2 ± 918.8.9	0.14
*Size birth (cm)*	51.7 ± 2.0	51.4 ± 2.0	0.73	51.5 ± 2.4	52.2 ± 2.0	0.38	53.0 ± 1.7	49.2 ± 0.8	0.02	52.5 ± 1.7	49.2 ± 0.8	*<0.05
*Neonate gender (% male)*	33.3	87.5		37.5	83.3		66.7	33.3		50	50	
*Delivery mode (% Cesarean)*	44.4	25		64.3	16.6		66.7	33.3		50	Accreta: 25 Increta:100 Percreta: 100	
*Gravida*	1.8 ± 1.1	1.8 ± 0.9	0.95	2.4 ± 1.6	2.1 ± 1.2	0.54	4.3 ± 3.0	3.7 ± 1.2	0.74	2.8 ± 2.9	4.3 ± 1.6	0.29
*Parity*	1.7 ± 0.9	1.5 ± 0.5	0.64	2.0 ± 1.4	1.7 ± 0.9	0.39	3.0 ± 2.0	1.3 ± 0.6	0.24	2.2 ± 1.9	2.9 ± 1.5	0.82
*PAS disorder*	None (9)	Accreta (8)		None (14)	Accreta (12)		None (3)	Accreta (3)		None (4)	Accreta (4) Increta (1) Percreta (1)	

Values are presented as the mean ± SD. NP, Normal pregnancy; PAS, placenta accreta spectrum. PCR Validation set includes samples of the initial set. *p<0.05 ^#^: Information of placenta weight is missing for placenta increta and percreta cases.

Placental tissue from Essen was obtained at the time of vaginal delivery or caesarian section from cases where PAS was diagnosed at the third stage of delivery or based on antepartum ultrasound measurements. Samples were classified into placenta accreta, increta or percreta (each n=1) according to the criteria defined by Cali et al. (6) and following the International Federation of Gynecology and Obstetrics (FIGO) guidelines based on intraoperative situation and histological findings (2). Intraoperatively, the area of placental tissue with the highest degree of invasion was chosen for analysis and was collected including surrounding tissue (decidua, myometrium, uterine serosa, broad ligament tissue). In the cases of placenta increta and percreta, the specimens were obtained by focal resection of the placenta or hysterectomy. Tissue samples were fixed in 4% formalin overnight followed by standard processing to obtain paraffin-embedded sections for ISH. The clinical characteristics are summarized in **Table 1**.

### 2.2 RNA isolation

Total RNA was isolated using a mirVana™ miRNA Isolation Kit (cat. No. AM1561; Invitrogen), according to the manufacturer’s protocol. Approximately 100 mg placenta tissue per sample was transferred to a Medicon (cat. No. 340591; BD Biosciences, Franklin Lakes NJ, USA) disposable for biological sample disaggregation containing 1 ml of lysis buffer (provided in the kit) and processed in a Medimachine (Dako; BD) for 20 s. Tissue suspension was collected, and Total RNA concentration was determined in a high-speed microfluidic UV/VIS spectrophotometer (QIAxpert System, Qiagen Hilden, Germany). Samples with A260/A280 ratio >1.8 were stored at −80°C until further processing.

### 2.3 Next-generation sequencing

GATC Biotech AG, Konstanz, Germany, performed the next-generation sequencing. The small RNA libraries were created using Illumina’s small RNA sample preparation protocol (TruSeq Small RNA Sample Prep Kits; Illumina, San Diego, CA, USA) with minor adaptations to the manufacturer’s instructions. Single read sequencing of the libraries was performed on a HiSeq 2500 (Illumina) according to the manufacturer’s protocol. At least 10 million reads per sample were generated.

### 2.4 Small RNA-Seq library processing, mapping, and differential expression analysis

First, the RA2 adapter sequences (5’-TGGAATTCTCGGGTGCCAAGG) of the TruSeq small RNA preparation kit were clipped from all reads, using cutadapt (25) (version 2.0), and all reads shorter than 15 bp or with a mean quality lower than 20 were removed subsequently. Read quality was monitored using FastQC (v0.11.3; http://www.bioinformatics.babraham.ac.uk/projects/fastqc). Quality reports of the raw and processed RNA-Seq libraries can be found at https://osf.io/8wq9h. Mapping was performed using TopHat2 (26)(version 2.1.1) with standard parameters onto the human reference genome (Ensembl release 98), and read counting was done using the respective Ensembl gene annotation. For counting, featureCounts (27) (version 1.6.3) with the parameters -M, -O 0.5 was used to count reads for the mature miRNA annotations of each human pre-miRNA separately. Analysis of differentially expressed miRNAs (DEmiRNAs), as well as plotting PCA, was performed by the Bioconductor R package DESeq2 (28) (version 1.10.0). Multiple testing adjustment of the resulting p-values was performed using Benjamini and Hochberg’s FDR approach (29). Mature miRNA loci with an identified adjusted p-value < 0.05 were considered differentially expressed. RNA-Seq datasets including DEmiRNA results and the count values are available at NCBI’s GEO database https://www.ncbi.nlm.nih.gov/geo/ under the accession IDGSE216742.

### 2.5 Biological pathway analysis and interaction network of miRNA targets

Target mRNAs of DEmiRNAs were obtained from the miRTarBase (30) (release 8.0) to get a non-redundant list of experimentally verified genes being potentially altered in PAS. Following the strategy used in our earlier study (31), DEmiRNA targets were assigned within the human regulatory pathways of the KEGG database. KEGG pathways were ranked individually according to the number of targeted genes within each pathway. The hypergeometric test was used to assess if specific pathways were significantly targeted by calculating the corresponding p-values for each pathway. Analysis of sub-pathways was performed by manually identifying key altered regions. To estimate which of the genes of the sub-pathway were most likely affected by the altered DEmiRNAs in PAS, an impact score was calculated based on the frequencies of the genes within the enriched pathways and the amount of DEmiRNAs targeting each gene.

### 2.6 Confirmation of DEmiRNAs

Expression levels of representative miRNAs were analyzed using the TaqMan™ Advanced miRNA cDNA Synthese-Kit (cat. No. A28007, Applied Biosystems, Darmstadt, Germany) and the TaqMan™ Fast Advanced Master Mix, no UNG (cat. No. A44360, Applied Biosystems) according with the manufacturer’s protocol with specific miRNA probes (hsa-miR-193b-3p, Assay ID: 478314_mir; hsa-miR-519d-3p, Assay ID: 478986_mir; hsa-miR-331-3p Assay ID: 478323_mir; hsa-miR-3074-5p Assay ID: 479606_mir; hsa-miR-24-3p Assay ID: 477992_mir; hsa-miR-382-3p Assay ID: 479458_mir; hsa-miR-376c-3p Assay ID: 478459_mir; hsa-miR-495-3p Assay ID: 478136_mir; hsa-miR-370-3p Assay ID: 478326_mir; hsa-miR-423-3p Assay ID: 478327_mir; hsa-miR-222-3p Assay ID: 477982_mir; hsa-miR-106b-3p Assay ID: 477866_mir; hsa-miR-4732-3p Assay ID: 478118_mir; hsa-miR-454-5p Assay ID: 478919_mir; hsa-mir-3615-3p Assay ID: 478837_mir; hsa-miR-16-2-3p Assay ID: 477931_mir; hsa-miR-39-3p Assay ID: 478293_mir). The *Caenorhabditis elegans* miRNA cel-miR-39 (Assay ID: 000200; 5′-UCACCGGGUGUAAAUCAGCUUG) was added at a concentration of 1.6 x 10^8^ copies/μL and used as spike-in control. PCR reactions were run in duplicates including no-template controls in 96-well plates on a Mx3005P qPCR System (Applied Biosystems) using 40 cycles, at the following conditions: 95°C for 3 sec and anneal/extend at 60°C for 20 sec. Fold changes were calculated by the formula 2^–ΔCt^ using cel-miR-39 as normalizer.

### 2.7 *In situ* localization of DEmiRNA

Representative miRNAs were localized in the placenta tissue by using the microRNA *in situ* hybridization (ISH) Buffer Set for formalin-fixed paraffin-embedded (FFPE) tissue samples (cat. No. 339457; Qiagen). Specific miRCURY LNA™ microRNA detection probes (cat. No. 339501; Qiagen), as well as positive and negative controls, were purchased from Qiagen (cat. No 339451). The one-day microRNA ISH protocol was carried out according to the supplier’s recommendations. In brief, paraffin blocks were cut into 6 μm-thick sections. Slides were dewaxed in a train of different percentages of xylene and ethanol solutions ending in phosphate-buffered saline (PBS) (cat No. 14190-094; Gibco, Schwerte, Germany). Following, slides were incubated with Proteinase-K for 10 min at 37°C in a CytoBrite Duo slide incubation system (SciGene; Sunnyvale, CA, USA) and then washed twice with PBS. Hybridization mix containing 10 nM of double-DIG LNA™ microRNA probe (miR-519d-3p, miR-193b-3p, miR-106b-3p, miR-370-3p or the negative control scramble probe SCR, which represents random sequence) was added to the slides and hybridized for 1 h. Slides were then washed in a slide rack with different concentrations of 5xSSC buffer (cat. No. 15557-044; Invitrogen) and placed in PBS. A hydrophobic barrier was created around the tissue sections using a Dako-Pen (Cat. No. H-4000; Vector Laboratories, Newark, CA, USA), and slides were incubated in a humidifying chamber with a blocking solution for 15 min. The blocking solution was removed, anti-DIG reagent (cat. No. 11093274910; Sigma Aldrich; Taufkirchen, Germany) was applied on the slides for 60 min incubation at RT. Sections were incubated with freshly prepared alkaline phosphatase substrate (cat. No. 11697471001; Merck, Darmstadt, Germany) for 2 h at 30°C, protected from light in the humidifying chamber. The reaction was stopped by incubating slides in KTBT buffer (Potassium-Tris Buffer with Triton). Nuclear Fast Red™ (cat. No. H-3403; Vector Laboratories) was applied for 1 min for nuclear counterstaining. Slides were dehydrated in ethanol solutions and mounted with 1-2 drops of mounting medium (cat. No. 03989; Sigma Aldrich), avoiding air-drying. The precipitate was allowed to settle overnight, and slides were analyzed using an Axio Imager A2 microscope and Zen Blue software (Carl Zeiss Microscopy GmbH, Jena Germany).

### 2.8 Expression of target mRNAs

Total RNA (300 ng) from NP and placenta accreta samples PAS was used to analyze the expression of selected mRNAs by reverse transcription using High-Capacity RNA-to-cDNA™ Kit (cat. No. 4368814; Applied Biosystems). Quantitative real-time PCR was performed using TaqMan assays (ERK1, Assay ID: Hs00385075_m1; NFKB1, Assay ID: Hs00765730_m1; AKT1, Assay ID: Hs00178289_m1; PTEN, Assay ID: Hs02621230_s1; STAT3, Assay ID: Hs00374280_m1; TGFB1, Assay ID: Hs00171257_m1; and GAPDH, Assay ID: Hs03929097_g1) and TaqMan Universal PCR Master Mix reagents (cat. No. 4440040; Applied Biosystems). qPCR was run on a Mx3005P qPCR System (Applied Biosystems). mRNA expression was normalized using the 2^−ΔCt^ method relative to GAPDH.

### 2.9 Immunofluorescence staining

Paraffin-embedded tissue sections were deparaffinized, hydrated in a graded ethanol series, and quenched by antigen retrieval with a citrate buffer (10 mM Sodium citrate, 0.05% Tween 20, pH 6.0) at >95° C for 10 min. Tissue sections were blocked with 0.1% BSA for 20 min and incubated with the primary antibodies mouse-anti-cytokeratin-7 (cat. no. MA1-06316; Invitrogen), rabbit-anti-PTEN (cat. No. 9559S; Cell Signaling, Danvers, MA, USA), and rabbit-anti-NF-κB (cat. No. SC-109; Santa Cruz Biotechnology, Heidelberg, Germany) for 2 h at 37°C in a humid atmosphere followed by incubation with the secondary antibodies goat anti-mouseAF488 (cat. No. A11017; Invitrogen) or goat anti-rabbitAF647 (cat. No. A21246; Invitrogen). All antibodies diluted 1:200 were applied and incubated 1 h at 37°C under humidity. DAPI (1 µg/mL) (cat. No. D9542; Sigma Aldrich) was used for nuclei staining. Fluorescence was visualized and recorded using a Zeiss LSM 710 confocal laser scanning microscope (Carl Zeiss Microscopy GmbH).

### 2.10 Statistical analysis

Unpaired Student t-test with Mann-Whitney test was applied to assess differences between groups using Prism software version 9 (GraphPad, San Diego, CA) as indicated at every figure legend. A p-value < 0.05 was considered significant.

## 3 Results

### 3.1 Identification of DEmiRNA in placenta accreta by high-throughput small RNA-Seq

**Figure 1:**
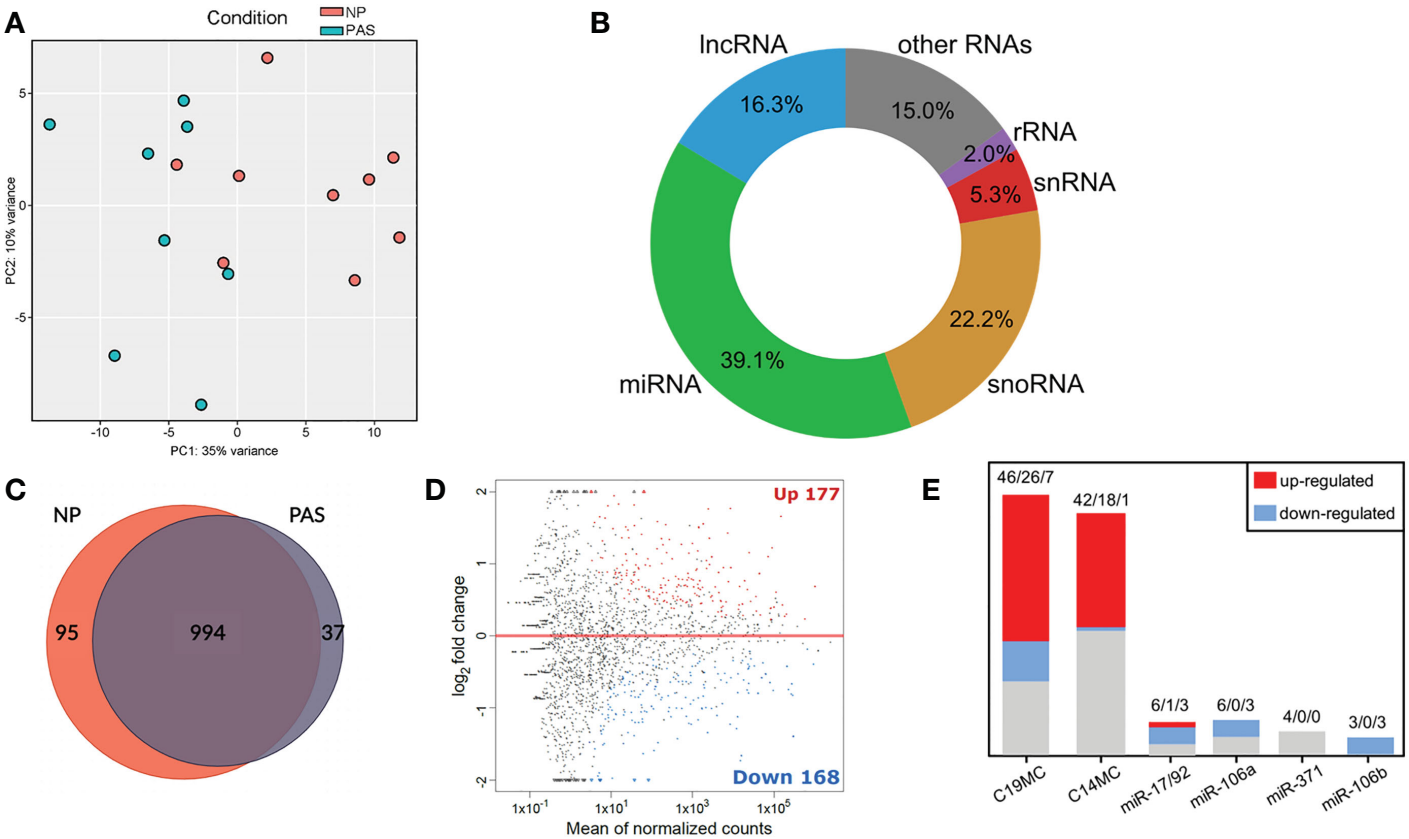
Small RNA-Seq analysis reveals a distinct expression pattern of miRNAs in placenta accreta samples. **(A)** PCA of the investigated samples based on all detected miRNAs. **(B)** Mapped sRNA reads were sorted into RNA classes. **(C)** Overlap of the actively transcribed miRNA genes in NP and PAS samples. **(D)** MA plot showing mature DEmiRNAs in PAS relative to NP. The x-axis is the log2 average expression over all samples, and the y-axis is the log2 fold change between PAS and NP groups. Red and blue dots represent respectively the significant differentially up- and down-expressed miRNAs. **(E)** Number of DEmiRNAs in PAS that belong to placental miRNA clusters. Numbers indicate total miRNA species in the cluster/miRNAs upregulated/miRNAs downregulated. NP, Normal pregnancy; PAS, Placenta Accreta Spectrum; C19MC, chromosome 19 miRNA cluster; C14MC, chromosome 14 miRNA cluster.

Normal (NP; n=9) and adherent accreta (PAS; n=8) placentas were analyzed by sRNA-Seq. At least 10 million reads per sample were obtained and used for library processing and mapping. Principal component analysis (PCA) revealed a separation of samples belonging to NP and PAS groups with some overlaps (**Figure 1A**). Most small RNA molecules were identified as miRNA species (39.9%), followed by small nucleolar RNAs (snoRNA; 22.2%) and long non-coding RNAs (lncRNA; 16.3%). A minor proportion included small nuclear RNAs (snRNA; 5.3%) and ribosomal RNA (rRNA; 2.0%) (**Figure 1B**). To identify the significant genes in PAS (p< 0.05), the DESeq2 R package was used. Placental tissues of NP and PAS shared a common miRNA signature consisting of 994 active miRNAs. Exclusively expressed were 95 miRNA species in NP and 37 in PAS placentas (**Figure 1C**). A total of 147 mature miRNAs were up- and 151 were downregulated in PAS compared to NP (**Figure 1D**). A selective analysis of miRNAs (17, 32–34) revealed DEmiRNAs in the placenta-associated clusters including the chromosome 19 miRNA cluster (C19MC; 33 out of 46 miRNA species in the cluster), the chromosome 14 miRNA cluster (C14MC; 19 out of 42), the miR-17/92 cluster (4 out of 6), the miR-106a cluster (3 out of 6), and the miR-106b cluster (3 out of 3), but not the miR-371 cluster (**Figure 1E**). A full list of miRNAs included in the clusters is presented in **Supplementary Table. 1**.

### 3.2 Validation of DEmiRNAs in placenta accreta

**Figure 2:**
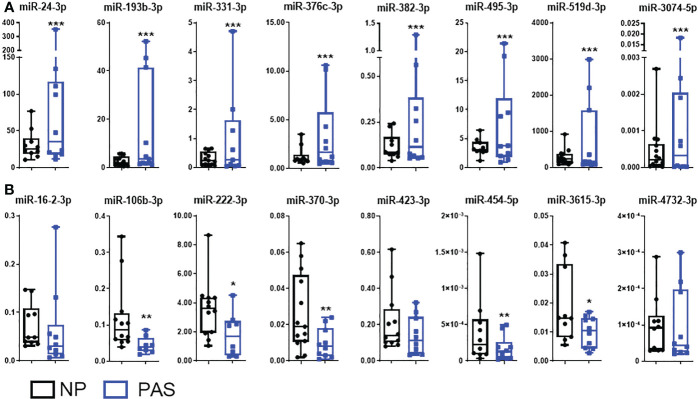
Validation of DEmiRNAs in placenta accreta samples. Expression patterns of differentially expressed miRNAs (DEmiRNAs) identified by RNA-Seq were validated by qRT-PCR in a larger sample cohort NP (n = 14) and PAS (n = 12). **(A)** Upregulated miRNAs and **(B)** downregulated miRNAs. The relative expression of each unique miRNA was normalized to the value of the exogenous cel-miR-39 using the 2^-ΔCt^ formula. Data are shown as the mean ± SE. Significant differences were determined by unpaired t- and Mann-Whitney test. ***p < 0.001, **p < 0.01, *p < 0.05.

DEmiRNAs were sorted according to the adjusted p-value, and a group of 16 DEmiRNAs exhibiting fold-change > 2.0, and good abundance (base mean > 100) were selected for individual validation using RT-qPCR in a larger cohort of samples (NP:14; Placenta accreta PAS:12) that includes the ones used for RNA-Seq. In this group, members of the C19MC (miR-519d-3p) and C14MC (miR-370-3p and miR-454-5p), as well as miRNAs not reported in PAS were included. Small RNA-Seq data was successfully validated in eight out of eight selected upregulated miRNAs in PAS samples: miR-24-3p, miR-193b-3p, miR-331-3p, miR-376c-3p, miR-382-3p, miR-495-3p, miR-519d-3p and miR-3074-5p (**Figure 2A**). Among downregulated miRNAs in PAS, five out of eight miRNA species were validated by RT-PCR (miR-106b-3p, miR-222-3p, miR-370-3p, miR-454-5p, and miR-3615-3p (**Figure 2B**).

### 3.3 DEmiRNAs localize mainly in trophoblast but also in endothelial cells

**Figure 3:**
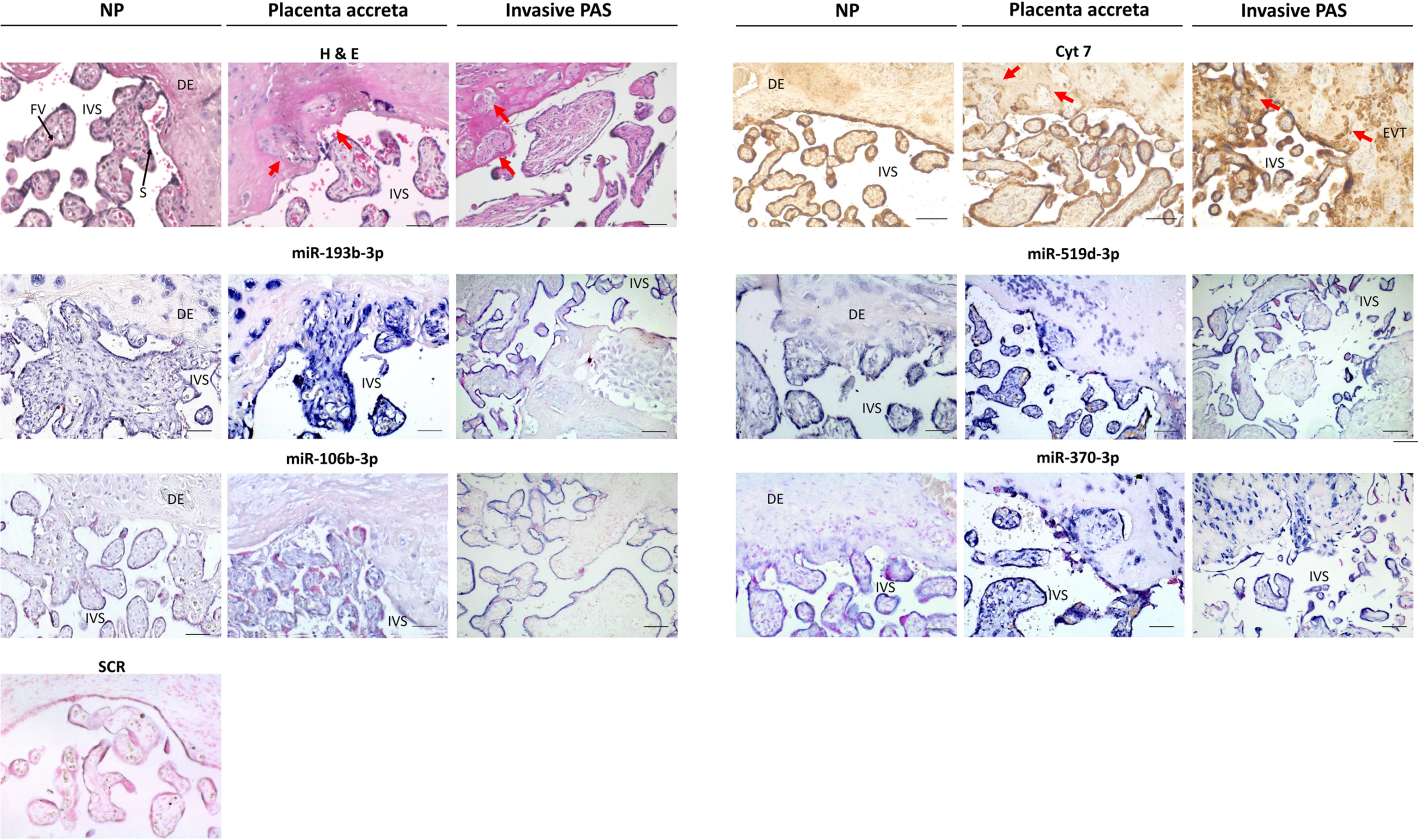
Differentially expressed miRNAs visualized by *in situ* hybridization in normal placenta (NP), placenta accreta, and placenta increta and percreta samples (invasive PAS). Areas containing villi and uterine tissue have been selected, in placenta accreta and invasive PAS with implanted villi and extravillous trophoblast cells (EVTs). The hematoxylin and eosin (H&E) staining shows nuclei in blue, cytoplasm in pink; cytokeratin-7 (CK7) staining marks positive cells brown (mainly syncytiotrophoblast and trophoblast cells) red arrows show areas of deep trophoblast infiltration; *in situ* hybridization of miR-193b-3p, miR-519d-3p, miR-106b-3p, and miR-370-3p shows positive cells in blue. Sections were counterstained with Nuclear Red. IVS, intervillous space; DE, decidua; FV, fetal vessel; S, Syncytiotrophoblast. Scale bar: 100 µm.

To determine the possibility of cell-specific expression, localization of DEmiRNAs in PAS was examined by ISH within placental villous tissue (**Figure 3**). Tissue sections of placenta accreta, placenta increta and percreta were stained with hematoxylin and eosin (H&E) to visualize morphological differences. Abnormally deep anchoring of the placental villi, as well as fibrin and trophoblast cells invaded into decidual tissue, were present in PAS samples (**Figure 3**). According to CK7 expression by IHC, extravillous trophoblast cells (EVTs) deeply infiltrating the decidual tissue were often observed in invasive PAS but not in NP samples (red arrows in **Figure 3**). To investigate the location of DEmiRNAs in the tissue, *in situ* hybridization was performed using digoxigenin-labeled LNA probes, which bind specifically to their target miRNA or that contain a random non-genomic scramble sequence (SCR) as negative control. ISH revealed miR-193b-3p signal in STB of both PAS and NP placentas, elevated miR-193b-3p expression was observed in PAS compared to NP samples, especially in the EVTs and areas of trophoblast invasiveness into the decidual tissue. The expression of miR-519d-3p, a placenta-specific miRNA, was restricted to trophoblast cells and strongly present on invasive trophoblast cells of PAS tissue. In NP tissue, the expression of miR-106b-3p and miR-370-3p was found mainly delimited in trophoblast cells, although endothelial cells were also positive for miR-370-3p. In PAS, miR-370-3p was highly expressed by invasive trophoblast cells. Contrary to PCR results, a downregulation of miR-106b-3p and miR-370-3p was not observable in PAS compared to NP samples.

### 3.4 Biological pathway analysis identifies cell cycle and inflammation pathways as networks of DEmiRNAs in placenta accreta

**Figure 4:**
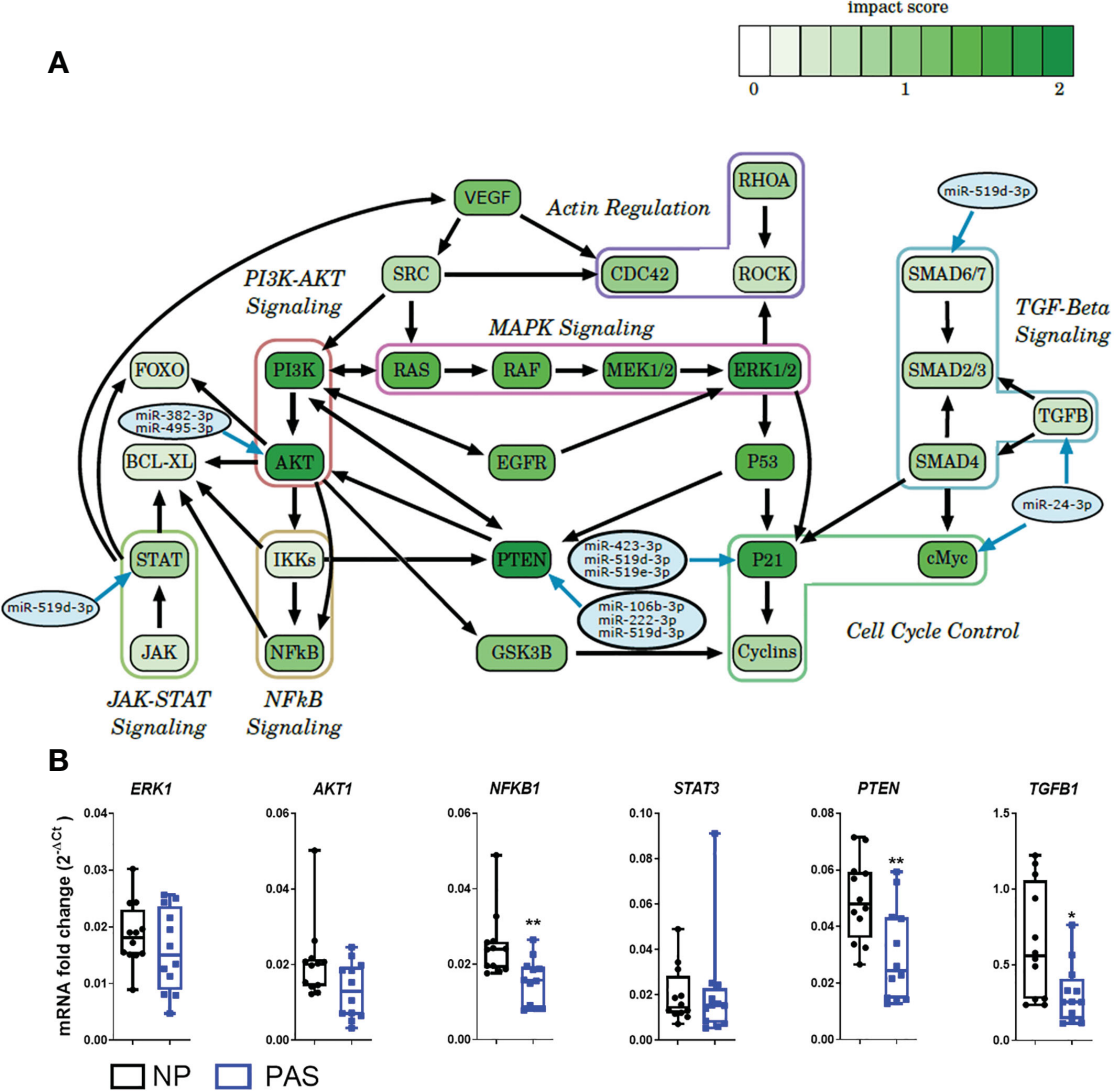
Expression of potential DEmiRNA targets in PAS. **(A)** Enriched pathways found in the KEGG analysis and putative DEmiRNAs targeting components of the pathway. The score assigned to each gene roughly describes the probability that this gene is regulated by the DEmiRNAs. The higher the score, the greater the influence of the DEmiRNAs on the gene within the pathway. **(B)** Expression of DEmiRNA mRNA targets by qRT-PCR in NP (n = 14) and PAS placentas (n = 12). The relative expression of each unique mRNA was normalized using the formula 2^-ΔCt^ with GAPDH as endogenous control. Data are shown as the mean ± SE. Significant differences were determined by the unpaired t- and Mann-Whitney test. *P < 0.05, **P < 0.01..

To explore alterations in gene expression, a hypergeometric test was run to identify KEGG pathways that the identified DEmiRNAs could alter. This analysis is based on the number of genes involved in each pathway which the DEmiRNAs can potentially regulate. As a result, 87 potentially altered pathways were identified. An additional inspection of these pathways allowed the identification of 7 shared sub-pathways, including cell cycle control, actin regulation, TGF-β, MAPK, PI3K-AKT, NF-κB, and the JAK-STAT signaling pathways (**Figure 4A**). A representative gene from each pathway was selected for validation by PCR. No significant expression difference was found for ERK1 and AKT mRNA (MAPK and PI3K-AKT signaling pathways are targeted by miR-382-3p and miR-495-3p), but a significant reduction of NF-κB mRNA was confirmed in PAS samples. As a representative of the JAK-STAT pathway with high invasion-inducing capacities (35), STAT3 expression was investigated but was not altered in PAS. Among the TGF-β pathway, TGF-β1 was downregulated in PAS samples. PTEN mRNA, which is involved in the cell cycle control pathway and a potential target of miR-106b-3p, miR-222-3p, and miR-519d-3p (36), was also decreased in PAS compared to NP samples (**Figure 4B**).

### 3.5 PTEN is down- while NF-κB is upregulated in placenta accreta

**Figure 5:**
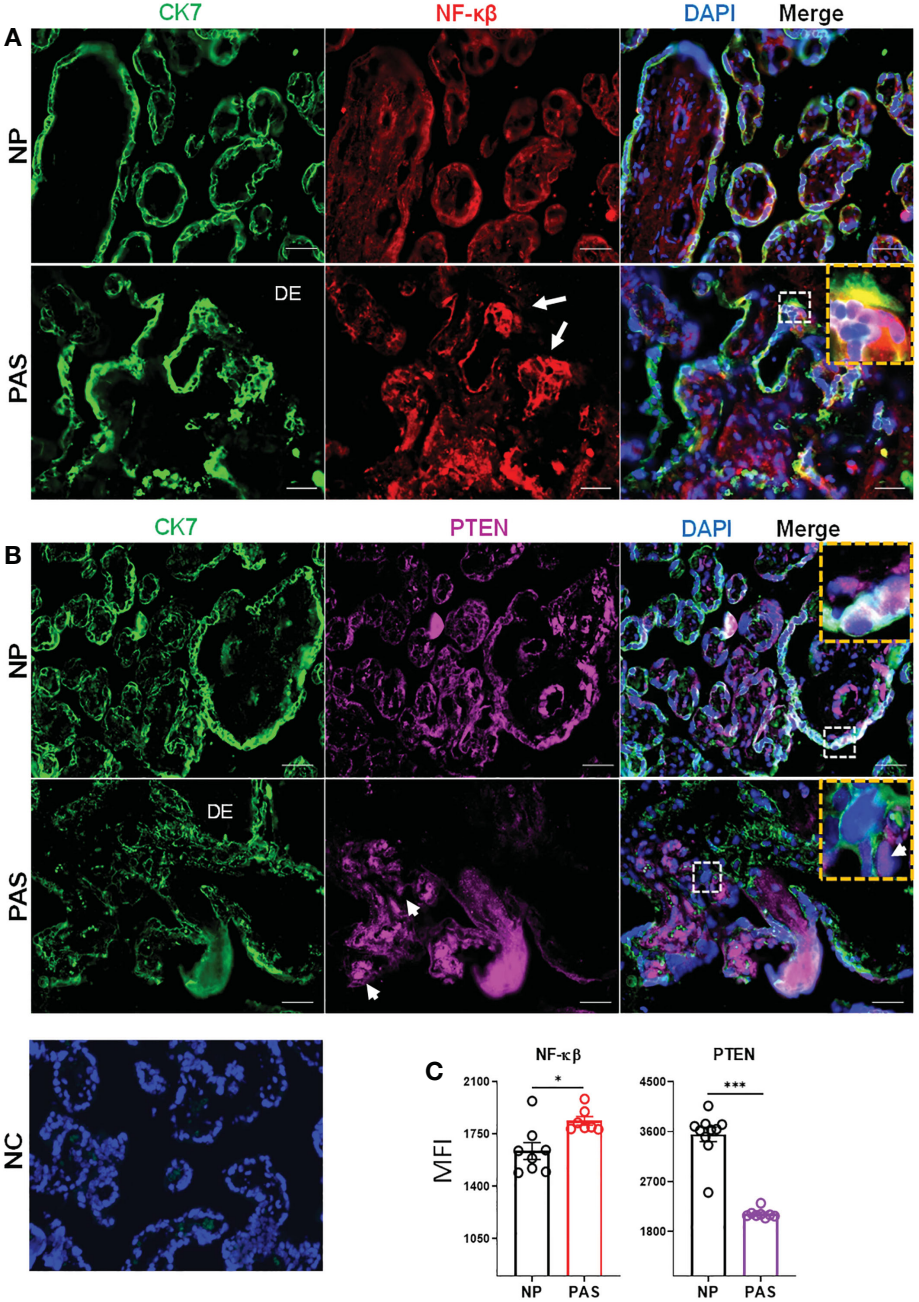
Immunolocalization of NF-κB and PTEN in normal and placenta accreta samples. Double immunolabelling of cytokeratin-7 (CK7, pseudo-green) with **(A)** NF-κB (pseudo-red) or **(B)** PTEN (pseudo-violet). Yellow boxes show the zoom-in area of white dotted boxes. White arrows and arrowheads indicate respectively invasive trophoblast columns and potential hofbauer cells. The scale bar represents 100 µm. **(C)** Extracted fluorescent intensity from representative pictures (n=3-5) in slides of NP (n=3) and PAS (n=3). Significant differences were determined by unpaired t- and Mann-Whitney test ***p < 0.001, *p < 0.05. DE, Decidua; NC, Negative control; NP, normal pregnancy; PAS, placenta accreta spectrum.

Based on the network of signaling pathways described above, two main transcription factors were selected for further investigation: PTEN, which is involved in cell cycle functions including proliferation, migration, and metabolism (37), and NF-κB, which is involved in the expression of inflammatory factors (38). To localize these proteins in placental tissues from NP and placenta accreta PAS samples, double immunofluorescence staining was carried out as described in the method section. In NP, the placenta villi appeared well delimitated by CK7 positive STB as observed in IHC staining. In contrast, PAS tissue showed zones with unorganized STB and the presence of large areas of EVT infiltration in the decidual tissue (**Figure 5**). In NP and PAS placenta, NF-κB was expressed in STB and the stroma of placental villi. Additionally, focal expression in areas of column-like EVTs was also observed in PAS (**Figure 5A**, white arrow). Contrary to the mRNA analysis, the fluorescence intensity of NF-kB protein was higher in PAS than NP (**Figure 5C**). In NP, PTEN was localized mainly in STB, endothelial cells surrounding fetal blood vessels, and in minor proportion in the stroma. Contrarily, in PAS samples, PTEN localized mainly in the stroma, partially in Hofbauer cells (white arrowheads), and in minor proportion in STB and EVTs (**Figure 5B**). In agreement with the mRNA validation, PTEN protein expression was reduced in PAS compared to NP tissues (**Figure 5C**).

### 3.6 Regulation of trophoblast invasion and migration are the most common functions of DEmiRNAs in PAS

To clarify the function of DEmiRNAs, literature was screened for investigations in physiological or pathological pregnancies. Among the confirmed DEmiRNAs, only miR-495-3p has not been reported as differentially expressed in any pathological pregnancy. Several DEmiRNAs have been previously identified altered in preeclampsia (PE), pre-term birth (PTB), fetal growth restriction (FGR), or gestational diabetes mellitus (GDM). To the best of our knowledge, no reports on PAS regarding the here described DEmiRNAs exist. Target genes confirmed in this study (e.g., TGF-β, MAPK, PETN) have been reported as validated targets of DEmiRNAs in other studies supporting the network associations proposed here. Most of the found studies described miRNA functions in trophoblast cells, and reported their association with cell migration, proliferation and epithelial-mesenchymal transition (**Table 2**).

**Table 2:** Previous reports of DEmiRNAs in PAS.

miRNA	Chr. Location	Expression in healthy and pathological pregnancy	Target gene(s)	Reported function	Reference
**miR-24-3p**	chr9: 95,086,064-95,086,085 chr19: 13,836,289-13,836,310	Upregulated in PE	TGF-β, MAPK, CDK, PI3K, p85, MYC, MM14	Regulation of actin organization. Cell migration and proliferation.	(39, 40)
		Upregulated in PPROM and PTB			(41)
**miR-193b-3p**	chr16: 14,304,017-14,304,038	Upregulated in PE	TGF-β2	Promotes trophoblast (HTR-8/SVneo) cell motility, migration and motion	(42–44)
		Upregulated in FGR			(45)
**miR-331-3p**	chr12: 95,308,420-95,308,513	Downregulated in PE	TGF-βR1	Regulates the invasion of human trophoblastic HTR-8/SVneo cells	(46, 47)
**miR-376c-3p**	chr14: 101,039,732-101,039,752 (miR-379/miR-656 cluster)	Upregulated at term labor	PHLDA2, HBEGF, TGF-β1R (ALK5 and ALK7)	Promotes trophoblast outgrowth and invasion	(48)
		Present in umbilical cord serum-derived exosomes			(49)
		Downregulated in PE			(50–52)
**miR-382-3p**	chr14: 101,054,352-101,054,372	Upregulated in PE	STAT1, NEAT1, ROCK1, PTEN	Inhibits cell proliferation and migration and its downregulation promotes invasiveness in cancer models	(53–55)
**miR-495-3p**	chr14: 101,033,804-101,033,825	Not reported yet			
**miR-519d-3p**	chr19: 53,713,400-53,713,421 (C19MC cluster)	Expressed almost exclusively in placenta tissue	CXCL6, NR4A2, FOXL2, PDCD4, PTEN, MMP-2	Reduces trophoblast cell migration and invasion Downregulates the EVT invasive phenotype	(56, 57)
		Upregulated in PE		Suppresses invasion and migration of trophoblast cells by targeting MMP-2	(58)
**miR-3074-5p**	chr9: 95,086,063-95,086,083	Upregulated in placental villi from recurrent miscarriage Low expressed in placental villi from NP	BCL2, FGF1, P27, BCL-G, DLST, GAP43, CCR3, RUNX2	Promotes apoptosis but inhibits invasion of HTR-8/SVneo cell line	(59, 60)
**miR-16-2-3p**	chr3: 160,404,797-160,404,818	Upregulated in placental villi and decidua from recurrent spontaneous abortion	VEGF	Regulates placental angiogenesis and development	(61)
		Upregulated in DICE-deficient HTR-8/SVneo trophoblast cell line	COL1A2	Reduces invasion of HTR-8/SVneo cell line	(62)
**miR-106b-3p**	chr7: 100,094,002- 100,094,023	Upregulated in PE	MMP-2	Inhibits the invasion and proliferation of JAR and JEG3 cells	(63)
**miR-222-3p**	chrX: 45,747,036-45,747,056	Upregulated in PE	BCL2L11	Promotes apoptosis of mesenchymal stem cells in response to hypoxia	(64)
			HDAC6	Inhibits trophoblast proliferation and migration	(65)
**miR-370-3p**	chr14: 100,911,186-100,911,207	Dysregulated in GTD Upregulated in FGR Downregulated in first-trimester healthy placenta		Involved in regulating proliferation, migration, and invasion of cancer cells	(19, 66, 67)
**miR-423-3p**	chr17: 30,117,131-30,117,153	Upregulated in early onset PE	MAPK signaling pathway		(68, 69)
**miR-454-5p**	chr17: 59,137,828-59,137,849	Downregulated in PE	ALK7	Promotes proliferation reduces apoptosis and increases invasion of trophoblast cells	(70)
**miR-3615-3p**	chr17: 74,748,663-74,748,683	Upregulated in plasma exosomes from PTB	TGF-β signaling	Possibly involved in trophoblast proliferation	(71)
**miR-4732-3p**	chr17: 28,861,668-28,861,688	Dysregulated in serum from PE Downregulated in GDM		Possibly involved in cellular development and cellular movement	(72, 73)

## 4 Discussion

The definition of PAS has been constantly revised in the last century because of the heterogeneous histological and clinical characteristics of deliveries complicated by placental retention. The currently recommended terminology includes different degrees of abnormal placentation from abnormally adherent villi towards extended EVT invasion into the uterine wall and beyond into adjacent organs (1). The incidence of this life-threatening disease is increasing rapidly, affecting at least 1 in 817 pregnancies worldwide and approximately 1 in 500 pregnancies in developed countries. A recent report indicates an incidence as high as 1 in 272 pregnancies (10).

In contrast to placenta accreta, the etiology of severe PAS (placenta increta/percreta) remains largely well defined since in nearly all cases a surgical damage of the decidua preceded. Currently, the most accepted hypothesis is a combination of scarred endometrium caused by damage prior to gestation and the subsequent abnormal invasion of trophoblast cells (74). Consequently, the most common risk factor for PAS is a history of cesarean deliveries and/or previous uterine surgeries (myomectomy, operative hysteroscopic procedures, dilation, and curettage, etc.), followed by assisted reproductive technology, especially *in vitro* fertilization and embryo transfer (IVF-ET) and advanced maternal age (75, 76). In our hands, the number of previous pregnancies was similar among the control and PAS groups, but all women with placenta increta or percreta presented previous uterine surgery. In contrast, several women included in the PAS group that suffered from adherent placenta accreta were primigravida, supporting the role of additional factors other than previous cesarean sections play a role in placenta accreta etiology.

Advanced clinical examinations, including ultrasound and in some cases also magnetic resonance imaging, may allow the diagnosis of severe PAS (placenta increta and percreta) with high sensitivity (88-97%) when used by skilled personnel (6). However, antenatal identification of adherent placenta accreta is limited and is reported as low as in only 33% of the cases (5). Making the diagnosis can be more challenging when patients are not considered at risk because they have no placenta previa or no history of previous uterine surgery (77). Likewise, these measures are insufficient to reliably predicting the exact extent of trophoblast invasion (78, 79). Consecutively, the final decision on the optimal method to deliver the placenta depends on the knowledge of the degree of placental invasion, that often only can be decided intraoperatively. Therefore, several authors attempted to identify biomarkers, including placental proteins (e.g., PAPP-A, AFP), hormones (e.g., hCG and human placenta lactogen), and, more recently, cell-free fetal DNA and cell-free placental mRNA, that could improve the accuracy of antenatal diagnosis of PAS [revised in (80, 81)]. Although these factors may be altered in PAS, there is a significant overlap with their concentration in unaffected pregnancies, which limits their applicability. Combining these with other markers such as miRNAs may potentially improve the diagnosis and clinical management of PAS. A deeper knowledge about the clinical behavior of PAS trophoblast cells may offer better surgical treatment or preventative procedures.

In the last years, miRNAs have been widely accepted as critical players in placental development. miRNA dysregulation is found in pregnancy complications such as preeclampsia (PE), early pregnancy loss, and fetal growth retardation (FGR) (82–84), but very few studies have sought to identify the miRNA expression profile in PAS. Here, we found that the placental expression of miRNAs differs between the adherent PAS and control groups. Although the statistical tool DESeq2 was initially designed to identify differential expression of mRNA and not miRNA genes, its basic model and normalization assumptions hold true for the investigated RNA-Seq datasets, e.g., most genes are not differentially expressed and there is a balance of over- and under-expression (28) (see **Figure 1D**). An independent study showed that DESeq2 could maintain a reasonable false-positive rate without a significant loss of power, even when executed on a dataset with a relatively low number of highly expressed genes, which is the case for most sRNA-Seq datasets (85). Using this strategy, a group of DEmiRNAs was identified which includes some members of the placenta-associated miRNA clusters C19MC, C14MC, miR-106a, miR-106b, and miR-17-92. These miRNA clusters regulate trophoblast functions, cell-cell communication, and are involved in viral infection responses and placental homeostasis (17, 32–34). In our hands, validation PCR for specific miRNAs carried out in a larger cohort of placenta accreta samples confirmed the differential expression of 13 miRNAs (8 upregulated and 5 downregulated), previously not reported concerning a role in PAS development. These miRNAs were localized by *in situ* hybridization revealing that they are expressed mainly by trophoblast cells and, in some cases, overexpressed by the invasive EVTs, especially observed in PAS placentas strengthening their role as regulators of trophoblast function. Remarkably, for ten of these miRNAs (miR-331-3p, -193b-3p, -376c-3p, -3074-5p, -222-3p, -519d-3p, -106b-3p, -3615, -16-2-3p und -454-5p, see **Table 2**), *in vitro* studies have already been carried out in trophoblastic cell lines, and they are reported to control trophoblast invasion and migration. Some of these miRNAs are also altered in PE, FGR, or other pregnancy disorders, suggesting their central role in placental functions and potentially a common alteration of trophoblast regulation in distinct pathologies. However, it cannot be ruled out that these miRNAs regulate different mRNAs to promote PAS development since there are certain redundancies and compensation effects among miRNAs (86). Likewise, the presence of DEmiRNAs considered of trophoblast origin (e.g., miR-519d-3p) in other cell types such as endothelial cells could indicate intercellular transfer from trophoblast to other placental cell types, which may cause alteration in the function of recipient cells. In the context of PAS, the relevance of this mechanism has not been addressed yet and could contribute to clarifying its etiology.

The majority of here identified miRNAs have been already tested in pregnancy-related pathologies at the placental level (**Table 2**), but researchers are now seeking to determine whether these miRNAs may serve as serum biomarkers. For instance, plasma miR-139-3p, miR-196a-5p, miR-518a-3p, and miR-671-3p were found downregulated in serum of patients diagnosed with placenta increta or percreta compared to healthy pregnancies (87). In our hands, miR-139-3p and miR-671-3p were also found downregulated in placenta accreta compared to NP placentas, which may support their use as biomarkers. However, miR-518a-3p appeared upregulated in our study. Likewise, the assessment of the secretory form of clusterin combined with the expression of either miR-21-5p, miR-92a-3p or miR-320a-3p in plasma of pregnant women were reported as potential predictors for the development of different forms of PAS with high specificity and sensibility (88). While no changes in miR-21-5p or miR-320a-3p were identified in our study, miR-92a-3p was found downregulated and not upregulated as suggested by that publication. Likewise, while our results showed increased miR-382-3p and decreased miR-423-5p expression in placenta tissue from adherent PAS, their serum levels appeared unchanged in the aforementioned studies (87, 88). Other miRNAs such as miR-24 and miR-519d were found here upregulated in PAS tissue and their plasma levels were found upregulated in other pathologies such as preeclampsia (89). Considering that placental miRNA expression changes with the gestation age (90) and the samples included in this study were taken after delivery, the low correlation with the reported alterations in plasma may be due to the differences in the gestational age at sampling. Therefore, although having the potential, more comprehensive studies are needed to determine whether the DEmiRNAs reported in this study may in fact serve as early diagnostic markers for PAS.

To further examine the biological relevance of DEmiRNAs in PAS, an *in silico* analysis was carried out to assign functional meaning for regulation at the mRNA level. To improve the interpretation of biological phenomena related to the extensive list of enriched KEGG pathways, analysis of local regions or sub-pathways has been achieved following a similar strategy as that published by others (91) and in our previous study (31). In the context of PAS, these bioinformatic strategies allowed the identification of biological pathways involved in angiogenesis, embryonic development, cell migration and adhesion, and tumor-related pathways that are deregulated in serum of PAS patients (87). Using a similar strategy, a network of lncRNAs, miRNAs and mRNAs implicated in reduced angiogenesis has been reported in PAS placentas (24). Here, seven sub-pathways, including cell cycle control, actin regulation, TGF-β, MAPK, PI3K-AKT, NF-κB, and the JAK-STAT signaling, were consistently mapped as targets of DEmiRNAs in the enriched KEGG pathways, which highlights them as the major cascades affected in PAS pathophysiology. Some molecules within these pathways have been previously reported as affected in PAS. For instance, an investigation by us reported increased mRNA and protein expression of cell cycle mediators (p21, p16, and CyclinD1) in PAS placentas compared to NP. However, this effect was reported to be significant only when delivery occurred after week 34, suggesting an additional temporary regulation (92). Other factors previously reported in PAS include TGF-β, which regulates cellular growth, motility, tumorigenesis, and trophoblastic EMT. It suppresses trophoblast invasion by regulating the transcription factors zinc finger protein SNAI (SNAIL) and Twist family basic helix-loop-helix transcription factor (TWIST) (93–95). In addition, silencing TGF-β type 1 receptor (TGFBR1) expression in trophoblastic cells significantly enhanced their trophoblastic invasiveness related to EMT promotion. Congruently, TGF-β negatively regulates trophoblast invasion by upregulating miR-7 in a SMAD2-dependent manner supporting the repression of EMT (96). Consistent with our findings, a significant decrease in relative TGF-β1 mRNA expression in tissue of PAS versus NP placenta has been reported (97). *TGFB* genes have been proposed in literature as targets of three DEmiRNAs identified in this study (miR-24-3p, miR-193b-3p and miR-3615-3p) (42) (71). Because miR-24-3p and miR-193b-3p were upregulated while miR-3615-3p was downregulated in PAS samples, it is unfeasible to estimate the contribution of each of these miRNAs to the overall decrease in TGF-β1 mRNA expression nor to the development of PAS. Therefore, these observations reinforce the need to consider larger miRNA:mRNA networks as causative of PAS rather than the association of a single miRNA and its targets reported *in vitro*.

Abnormal expression of other genes identified in sub-pathway analyses has been reported in pregnancy malignancies. For instance, dysregulated PTEN expression in blastocyst implantation, spontaneous abortion, and PE has been reported suggesting its critical role during pregnancy (98–101). Although NF-κB signaling is mainly involved in regulating inflammatory factors, there is evidence that it negatively regulates cell cycle and cell proliferation (102). Several studies have reported associations between PTEN and NF-κB. Increased PTEN, dependent on the AP-1/NF-κB pathway, impairs human trophoblast cell invasion and is related to PE development (103). Furthermore, PTEN has been shown to promote NF-κB activation or suppression in other cell systems (104, 105). In our study presented here, we have found downregulated PTEN and NFKB mRNA in placenta accreta samples, while PTEN protein was downregulated and NF-κB protein was upregulated (in EVTs). Previously, we have reported that overexpression of miR-519d-3p in trophoblast cell lines is related to AKT upregulation but PTEN downregulation. We found miR-519d-3p associated with augmented trophoblast proliferation but reduced migration (56). Here, PAS samples showed high expression of miR-519d-3p, reinforcing its link with the PTEN/AKT/NF-κB system, which constitutes a vital cell cycle signaling pathway involved in trophoblast proliferation and metabolism. PTEN has been demonstrated as a common target of numerous miRNAs, including miR-21, miR-214, and miR-217, which are involved in regulating several cancer types (106, 107). In our study, in addition to miR-519d-3p, PTEN appears to be a potential target of miR-222-3p and miR-106b-3p and AKT of miR-382-3p and miR-495-3p.

In summary, this study provides a set of miRNAs as potential biomarkers for the diagnosis of PAS, especially for placenta accreta. Additionally, these miRNAs and their targets are associated with signaling pathways relevant for controlling trophoblast function, providing preliminary evidence for their role in the pathogenesis of PAS.

## Data availability statement

The data presented in the study are deposited in the OSF (https://osf.io/) and NIH (https://www.ncbi.nlm.nih.gov/geo/) repositories, accession numbers: https://osf.io/8wq9h 2022-12-08 and GSE216742 2022-11-01, respectively.

## Ethics statement

The studies involving human participants were reviewed and approved by the ethics committees from the Jena University Hospital and the Department of Gynecology and Obstetrics, University Hospital Essen, Germany. Experiments in the study were approved according to the Helsinki Declaration on ethical principles for medical research involving human subjects by obtaining consent forms (Amendment No: 1509-03/05 Jena and 12-5212-BO Essen). The patients/participants provided their written informed consent to participate in this study.

## Author contributions

Conceptualization, DM-P and UM. Formal analysis, JM-C, EB, RG-S, and DM-P. Funding acquisition, UM and DM-P. Investigation, JM-C, EB, PF-Z and RG-S. Project administration, DM-P. Supervision, MM, UM and DM-P. Resources, TG, AG, AK und MM. Visualization, JM-C, EB, P-FZ, RG-S and DM-P. Writing—original draft. JM-C and DM-P. Writing—review and editing, JM-C, EB, TG, AG, AK, MM, UM and DM-P. All authors contributed to the article and approved the submitted version.

## References

[1] Hecht JL Baergen R Ernst LM Katzman PJ Jacques SM Jauniaux E. Classification and reporting guidelines for the pathology diagnosis of placenta accreta spectrum (PAS) disorders: recommendations from an expert panel. Mod Pathol (2020) 33(12):2382–96. doi: 10.1038/s41379-020-0569-1 32415266

[2] Jauniaux E Ayres-de-Campos D Langhoff-Roos J Fox KA Collins S. FIGO classification for the clinical diagnosis of placenta accreta spectrum disorders. Int J Gynaecol Obstet (2019) 146(1):20–4. doi: 10.1002/ijgo.12761 31173360

[3] Jauniaux E Collins S Burton GJ. Placenta accreta spectrum: pathophysiology and evidence-based anatomy for prenatal ultrasound imaging. Am J Obstet Gynecol (2018) 218(1):75–87. doi: 10.1016/j.ajog.2017.05.067 28599899

[4] Jauniaux E Chantraine F Silver RM Langhoff-Roos J. FIGO consensus guidelines on placenta accreta spectrum disorders: Epidemiology. Int J Gynaecol Obstet (2018) 140(3):265–73. doi: 10.1002/ijgo.12407 29405321

[5] Bluth A Schindelhauer A Nitzsche K Wimberger P Birdir C. Placenta accreta spectrum disorders-experience of management in a German tertiary perinatal centre. Arch Gynecol Obstet (2021) 303(6):1451–60. doi: 10.1007/s00404-020-05875-x PMC8087589 33284419

[6] Cali G Giambanco L Puccio G Forlani F. Morbidly adherent placenta: evaluation of ultrasound diagnostic criteria and differentiation of placenta accreta from percreta. Ultrasound Obstet Gynecol (2013) 41(4):406–12. doi: 10.1002/uog.12385 23288834

[7] Bailit JL Grobman WA Rice MM Reddy UM Wapner RJ Varner MW. Morbidly adherent placenta treatments and outcomes. Obstet Gynecol (2015) 125(3):683–9. doi: 10.1097/AOG.0000000000000680 PMC4347990 25730233

[8] Matsuzaki S Mandelbaum RS Sangara RN McCarthy LE Vestal NL Klar M. Trends, characteristics, and outcomes of placenta accreta spectrum: a national study in the united states. Am J Obstet Gynecol (2021) 225(5):534 e1–534 e38. doi: 10.1016/j.ajog.2021.04.233 33894149

[9] Jauniaux E Burton GJ. Pathophysiology of placenta accreta spectrum disorders: A review of current findings. Clin Obstet Gynecol (2018) 61(4):743–54. doi: 10.1097/GRF.0000000000000392 30299280

[10] Gynecologists A.C.o.O.a S.f.M.-F. Medicine. Obstetric care consensus no. 7: Placenta accreta spectrum. Obstet Gynecol (2018) 132(6):e259–75. doi: 10.1097/AOG.0000000000002983 30461695

[11] Jauniaux E Bunce C Grønbeck L Langhoff-Roos J. Prevalence and main outcomes of placenta accreta spectrum: a systematic review and meta-analysis. Am J Obstet Gynecol (2019) 221(3):208–18. doi: 10.1016/j.ajog.2019.01.233 30716286

[12] Wu S Kocherginsky M Hibbard JU. Abnormal placentation: twenty-year analysis. Am J Obstet Gynecol (2005) 192(5):1458–61. doi: 10.1016/j.ajog.2004.12.074 15902137

[13] Chuong EB Hannibal RL Green SL Baker JC. Evolutionary perspectives into placental biology and disease. Appl Transl Genom (2013) 2:64–9. doi: 10.1016/j.atg.2013.07.001 PMC5121266 27896057

[14] Zhou J Chen H Xu X Liu Y Chen S Yang S. Uterine damage induces placenta accreta and immune imbalance at the maternal-fetal interface in the mouse. Placenta (2022) 119:8–16. doi: 10.1016/j.placenta.2022.01.002 35066308

[15] Li N Hou R Liu C Yang T Qiao C Wei J. Integration of transcriptome and proteome profiles in placenta accreta reveals trophoblast over-migration as the underlying pathogenesis. Clin Proteomics (2021) 18(1):31. doi: 10.1186/s12014-021-09336-8 34963445 PMC8903580

[16] Liang Y Ridzon D Wong L Chen C. Characterization of microRNA expression profiles in normal human tissues. BMC Genomics (2007) 8:166. doi: 10.1186/1471-2164-8-166 17565689 PMC1904203

[17] Morales-Prieto DM Ospina-Prieto S Chaiwangyen W Schoenleben M Markert UR. Pregnancy-associated miRNA-clusters. J Reprod Immunol (2013) 97(1):51–61. doi: 10.1016/j.jri.2012.11.001 23432872

[18] Mayor-Lynn K Toloubeydokhti T Cruz AC Chegini N. Expression profile of microRNAs and mRNAs in human placentas from pregnancies complicated by preeclampsia and preterm labor. Reprod Sci (2011) 18(1):46–56. doi: 10.1177/1933719110374115 21079238 PMC3343068

[19] Morales-Prieto DM Chaiwangyen W Ospina-Prieto S Schneider U Herrmann J Gruhn B. MicroRNA expression profiles of trophoblastic cells. Placenta (2012) 33(9):725–34. doi: 10.1016/j.placenta.2012.05.009 22721760

[20] Gu Y Bian Y Xu X Wang X Zuo C Meng J. Downregulation of miR-29a/b/c in placenta accreta inhibits apoptosis of implantation site intermediate trophoblast cells by targeting MCL1. Placenta (2016) 48:13–9. doi: 10.1016/j.placenta.2016.09.017 27871464

[21] Umemura K Ishioka S Endo T Ezaka Y Takahashi M Saito T. Roles of microRNA-34a in the pathogenesis of placenta accreta. J Obstet Gynaecol Res (2013) 39(1):67–74. doi: 10.1111/j.1447-0756.2012.01898.x 22672425

[22] Gu Y Meng J Zuo C Wang S Li H Zhao S. Downregulation of MicroRNA-125a in placenta accreta spectrum disorders contributes antiapoptosis of implantation site intermediate trophoblasts by targeting MCL1. Reprod Sci (2019) 26(12):1582–9. doi: 10.1177/1933719119828040 30782086

[23] Li N Hou R Yang T Liu C Wei J. miR-193a-3p mediates placenta accreta spectrum development by targeting EFNB2 via epithelial-mesenchymal transition pathway under decidua defect conditions. Front Mol Biosci (2020) 7:613802. doi: 10.3389/fmolb.2020.613802 33585562 PMC7873918

[24] Yang T Li N Hou R Qiao C Liu C. Development and validation of a four-microRNA signature for placenta accreta spectrum: an integrated competing endogenous RNA network analysis. Ann Transl Med (2020) 8(15):919. doi: 10.21037/atm-20-1150 32953719 PMC7475428

[25] Martin M. Cutadapt removes adapter sequences from high-throughput sequencing reads. EMBnet.journal (2011) 17(1):3. doi: 10.14806/ej.17.1.200

[26] Kim D Pertea G Trapnell C Pimentel H Kelley R Salzberg SL. TopHat2: accurate alignment of transcriptomes in the presence of insertions, deletions and gene fusions. Genome Biol (2013) 14(4):R36. doi: 10.1186/gb-2013-14-4-r36 23618408 PMC4053844

[27] Liao Y Smyth GK Shi W. featureCounts: an efficient general purpose program for assigning sequence reads to genomic features. Bioinformatics (2014) 30(7):923–30. doi: 10.1093/bioinformatics/btt656 24227677

[28] Love MI Huber W Anders S. Moderated estimation of fold change and dispersion for RNA-seq data with DESeq2. Genome Biol (2014) 15(12):550. doi: 10.1186/s13059-014-0550-8 25516281 PMC4302049

[29] Benjamini Y Drai D Elmer G Kafkafi N Golani I. Controlling the false discovery rate in behavior genetics research. Behav Brain Res (2001) 125(1-2):279–84. doi: 10.1016/S0166-4328(01)00297-2 11682119

[30] Huang HY Lin YC Li J Huang KY Shrestha S Hong HC. miRTarBase 2020: updates to the experimentally validated microRNA-target interaction database. Nucleic Acids Res (2020) 48(D1):D148–54. doi: 10.1093/nar/gkz896 PMC7145596 31647101

[31] Morales-Prieto DM Barth E Murrieta-Coxca JM Favaro RR Gutiérrez-Samudio RN Chaiwangyen W. Identification of miRNAs and associated pathways regulated by leukemia inhibitory factor in trophoblastic cell lines. Placenta (2019) 88:20–7. doi: 10.1016/j.placenta.2019.09.005 31586768

[32] Malnou EC Umlauf D Mouysset M Cavaillé J. Imprinted MicroRNA gene clusters in the evolution, development, and functions of mammalian placenta. Front Genet (2018) 9:706. doi: 10.3389/fgene.2018.00706 30713549 PMC6346411

[33] Chen DB Wang W. Human placental microRNAs and preeclampsia. Biol Reprod (2013) 88(5):130. doi: 10.1095/biolreprod.113.107805 23575145 PMC4013914

[34] Ouyang Y Bayer A Chu T Tyurin VA Kagan VE Morelli AE. Isolation of human trophoblastic extracellular vesicles and characterization of their cargo and antiviral activity. Placenta (2016) 47:86–95. doi: 10.1016/j.placenta.2016.09.008 27780544 PMC5123854

[35] Fitzgerald JS Poehlmann TG Schleussner E Markert UR. Trophoblast invasion: the role of intracellular cytokine signalling via signal transducer and activator of transcription 3 (STAT3). Hum Reprod Update (2008) 14(4):335–44. doi: 10.1093/humupd/dmn010 18424427

[36] Chaiwangyen W Ospina-Prieto S Photini SM Schleussner E Markert UR Morales-Prieto DM. Dissimilar microRNA-21 functions and targets in trophoblastic cell lines of different origin. Int J Biochem Cell Biol (2015) 68:187–96. doi: 10.1016/j.biocel.2015.08.018 26320576

[37] Chen CY Chen J He L Stiles BL. PTEN: Tumor suppressor and metabolic regulator. Front Endocrinol (Lausanne) (2018) 9:338. doi: 10.3389/fendo.2018.00338 30038596 PMC6046409

[38] Liu T Zhang L Joo D Sun SC. NF-κB signaling in inflammation. Signal Transduct Target Ther (2017) 2:e17023. doi: 10.1038/sigtrans.2017.23 PMC5661633 29158945

[39] Luizon MR Conceição IMCA Viana-Mattioli S Caldeira-Dias M Cavalli RC Sandrim VC. Circulating MicroRNAs in the second trimester from pregnant women who subsequently developed preeclampsia: Potential candidates as predictive biomarkers and pathway analysis for target genes of miR-204-5p. Front Physiol (2021) 12:678184. doi: 10.3389/fphys.2021.678184 34630130 PMC8493119

[40] Wu L Zhou H Lin H Qi J Zhu C Gao Z. Circulating microRNAs are elevated in plasma from severe preeclamptic pregnancies. Reproduction (2012) 143(3):389–97. doi: 10.1530/REP-11-0304 22187671

[41] Hromadnikova I Kotlabova K Krofta L. A history of preterm delivery is associated with aberrant postpartal MicroRNA expression profiles in mothers with an absence of other pregnancy-related complications. Int J Mol Sci (2021) 22(8):4033. doi: 10.3390/ijms22084033 33919834 PMC8070839

[42] Zhou X Li Q Xu J Zhang X Zhang H Xiang Y. The aberrantly expressed miR-193b-3p contributes to preeclampsia through regulating transforming growth factor-beta signaling. Sci Rep (2016) 6:19910. doi: 10.1038/srep19910 26822621 PMC4731805

[43] Xu P Zhao Y Liu M Wang Y Wang H Li YX. Variations of microRNAs in human placentas and plasma from preeclamptic pregnancy. Hypertension (2014) 63(6):1276–84. doi: 10.1161/HYPERTENSIONAHA.113.02647 24664294

[44] Betoni JS Derr K Pahl MC Rogers L Muller CL Packard RE. MicroRNA analysis in placentas from patients with preeclampsia: comparison of new and published results. Hypertens Pregnancy (2013) 32(4):321–39. doi: 10.3109/10641955.2013.807819 23844600

[45] Ostling H Kruse R Helenius G Lodefalk M. Placental expression of microRNAs in infants born small for gestational age. Placenta (2019) 81:46–53. doi: 10.1016/j.placenta.2019.05.001 31138431

[46] Shan L Hou X. Circular RNA hsa_circ_0026552 inhibits the proliferation, migration and invasion of trophoblast cells via the miR3313p/TGFbetaR1 axis in preeclampsia. Mol Med Rep (2021) 24(5):798. doi: 10.3892/mmr.2021.12438 34523694 PMC8456345

[47] Yi Y Cheng JC Klausen C Leung PCK. TGF-beta1 inhibits human trophoblast cell invasion by upregulating cyclooxygenase-2. Placenta (2018) 68:44–51. doi: 10.1016/j.placenta.2018.06.313 30055669

[48] Yadava SM Feng A Parobchak N Wang B Rosen T. miR-15b-5p promotes expression of proinflammatory cytokines in human placenta by inhibiting apelin signaling pathway. Placenta (2021) 104:8–15. doi: 10.1016/j.placenta.2020.11.002 33197856

[49] Jia L Zhou X Huang X Xu X Jia Y Wu Y. Maternal and umbilical cord serum-derived exosomes enhance endothelial cell proliferation and migration. FASEB J (2018) 32(8):4534–43. doi: 10.1096/fj.201701337RR 29570394

[50] Li J Du J Wang Z Wang C Bai J Zhang S. Expression of miR-376 in blood of pregnant women with preeclampsia and its effect on 25-hydroxyvitamin d. Exp Ther Med (2018) 16(3):1701–6. doi: 10.3892/etm.2018.6394 PMC6122294 30186390

[51] Yang HL Zhang HZ Meng FR Han SY Zhang M. Differential expression of microRNA-411 and 376c is associated with hypertension in pregnancy. Braz J Med Biol Res (2019) 52(4):e7546. doi: 10.1590/1414-431x20197546 30970081 PMC6459465

[52] Fu G Ye G Nadeem L Ji L Manchanda T Wang Y. MicroRNA-376c impairs transforming growth factor-beta and nodal signaling to promote trophoblast cell proliferation and invasion. Hypertension (2013) 61(4):864–72. doi: 10.1161/HYPERTENSIONAHA.111.203489 23424236

[53] Zhang H Zhu C He Z Chen S Li L Sun C. LncRNA PSMB8-AS1 contributes to pancreatic cancer progression via modulating miR-382-3p/STAT1/PD-L1 axis. J Exp Clin Cancer Res (2020) 39(1):179. doi: 10.1186/s13046-020-01687-8 32891166 PMC7487636

[54] Liu Y Wang Y Fu X Lu Z. Long non-coding RNA NEAT1 promoted ovarian cancer cells' metastasis through regulation of miR-382-3p/ROCK1 axial. Cancer Sci (2018) 109(7):2188–98. doi: 10.1111/cas.13647 PMC6029817 29790629

[55] Wang J Luo J Liu G Li X. Circular RNA hsa_circ_0008285 inhibits colorectal cancer cell proliferation and migration via the miR-382-5p/PTEN axis. Biochem Biophys Res Commun (2020) 527(2):503–10. doi: 10.1016/j.bbrc.2020.03.165 32423803

[56] Chaiwangyen W Murrieta-Coxca JM Favaro RR Photini SM Gutiérrez-Samudio RN Schleussner E. MiR-519d-3p in trophoblastic cells: Effects, targets and transfer to allogeneic immune cells via extracellular vesicles. Int J Mol Sci (2020) 21(10):3458. doi: 10.3390/ijms21103458 32422900 PMC7278925

[57] Xie L Mouillet JF Chu T Parks WT Sadovsky E Knöfler M. C19MC microRNAs regulate the migration of human trophoblasts. Endocrinology (2014) 155(12):4975–85. doi: 10.1210/en.2014-1501 PMC4239420 25211593

[58] Ding J Huang F Wu G Han T Xu F Weng D. MiR-519d-3p suppresses invasion and migration of trophoblast cells via targeting MMP-2. PloS One (2015) 10(3):e0120321. doi: 10.1371/journal.pone.0120321 25803859 PMC4372600

[59] Gu Y Shi Y Yang Q Gu WW He YP Zheng HJ. miR-3074-5p promotes the apoptosis but inhibits the invasiveness of human extravillous trophoblast-derived HTR8/SVneo cells in vitro. Reprod Sci (2018) 25(5):690–9. doi: 10.1177/1933719117725823 28826362

[60] Gu Y Zhang X Yang Q Wang J He Y Sun Z. Aberrant placental villus expression of miR-486-3p and miR-3074-5p in recurrent miscarriage patients and uterine expression of these MicroRNAs during early pregnancy in mice. Gynecol Obstet Invest (2015) 81:112–7. doi: 10.1159/000435879 26278328

[61] Zhu Y Lu H Huo Z Ma Z Dang J Dang W. MicroRNA-16 inhibits feto-maternal angiogenesis and causes recurrent spontaneous abortion by targeting vascular endothelial growth factor. Sci Rep (2016) 6:35536. doi: 10.1038/srep35536 27748453 PMC5066269

[62] Tang L Yang M Qin L Li X He G Liu X. Deficiency of DICER reduces the invasion ability of trophoblasts and impairs the pro-angiogenic effect of trophoblast-derived microvesicles. J Cell Mol Med (2020) 24(9):4915–30. doi: 10.1111/jcmm.14917 PMC7205818 32198822

[63] Li J Wang JM Liu YH Zhang Z Han N Wang JY. [Effect of microRNA-106b on the invasion and proliferation of trophoblasts through targeting MMP-2]. Zhonghua Fu Chan Ke Za Zhi (2017) 52(5):327–32. doi: 10.3760/cma.j.issn.0529-567X.2017.05.007 28545271

[64] Qu HM Qu LP Pan XZ Mu LS. Upregulated miR-222 targets BCL2L11 and promotes apoptosis of mesenchymal stem cells in preeclampsia patients in response to severe hypoxia. Int J Clin Exp Pathol (2018) 11(1):110–9. PMC6957949 31938092

[65] Liu T Li W Zhang J Zhang Y. MiR-222-3p inhibits trophoblast cell migration and alleviates preeclampsia in rats through inhibiting HDAC6 and Notch1 signaling. Reprod Sci (2021) 29(5):1486–97. doi: 10.1007/s43032-021-00793-y 34796469

[66] Zhao JR Cheng WW Wang YX Cai M Wu WB Zhang HJ. Identification of microRNA signature in the progression of gestational trophoblastic disease. Cell Death Dis (2018) 9(2):94. doi: 10.1038/s41419-017-0108-2 29367697 PMC5833456

[67] Wen H Chen L He J Lin J. MicroRNA expression profiles and networks in placentas complicated with selective intrauterine growth restriction. Mol Med Rep (2017) 16(5):6650–73. doi: 10.3892/mmr.2017.7462 PMC5865797 28901463

[68] Lykoudi A Kolialexi A Lambrou GI Braoudaki M Siristatidis C Papaioanou GK. Dysregulated placental microRNAs in early and late onset preeclampsia. Placenta (2018) 61:24–32. doi: 10.1016/j.placenta.2017.11.005 29277268

[69] Vashukova ES Glotov AS Baranov VS. MicroRNAs associated with preeclampsia. Russian J Genet (2020) 56(1):1–16. doi: 10.1134/S1022795419080167

[70] Shi Z She K Li H Yuan X Han X Wang Y. MicroRNA-454 contributes to sustaining the proliferation and invasion of trophoblast cells through inhibiting Nodal/ALK7 signaling in pre-eclampsia. Chem Biol Interact (2019) 298:8–14. doi: 10.1016/j.cbi.2018.10.012 30367833

[71] Menon R Debnath C Lai A Guanzon D Bhatnagar S Kshetrapal PK. Circulating exosomal miRNA profile during term and preterm birth pregnancies: A longitudinal study. Endocrinology (2019) 160(2):249–75. doi: 10.1210/en.2018-00836 PMC6394761 30358826

[72] Srinivasan S Treacy R Herrero T Olsen R Leonardo TR Zhang X. Discovery and verification of extracellular miRNA biomarkers for non-invasive prediction of pre-eclampsia in asymptomatic women. Cell Rep Med (2020) 1(2):100013. doi: 10.1016/j.xcrm.2020.100013 32864636 PMC7455024

[73] Ding R Guo F Zhang Y Liu XM Xiang YQ Zhang C. Integrated transcriptome sequencing analysis reveals role of miR-138-5p/ TBL1X in placenta from gestational diabetes mellitus. Cell Physiol Biochem (2018) 51(2):630–46. doi: 10.1159/000495319 30463081

[74] Jauniaux E Jurkovic D Hussein AM Burton GJ. New insights into the etiopathology of placenta accreta spectrum. Am J Obstetrics Gynecology (2022) 227(3):384–91. doi: 10.1016/j.ajog.2022.02.038 35248577

[75] Eshkoli T Weintraub AY Sergienko R Sheiner E. Placenta accreta: risk factors, perinatal outcomes, and consequences for subsequent births. Am J Obstet Gynecol (2013) 208(3):219.e1–7. doi: 10.1016/j.ajog.2012.12.037 23313722

[76] Baldwin HJ Patterson JA Nippita TA Torvaldsen S Ibiebele I Simpson JM. Antecedents of abnormally invasive placenta in primiparous women: Risk associated with gynecologic procedures. Obstet Gynecol (2018) 131(2):227–33. doi: 10.1097/AOG.0000000000002434 29324602

[77] Comstock CH. Antenatal diagnosis of placenta accreta: a review. Ultrasound Obstet Gynecol (2005) 26(1):89–96. doi: 10.1002/uog.1926 15971281

[78] D'Antonio F Palacios-Jaraquemada J Timor-Trisch I Cali G. Placenta accreta spectrum disorders: Prenatal diagnosis still lacks clinical correlation. Acta Obstet Gynecol Scand (2018) 97(7):773–5. doi: 10.1111/aogs.13374 29924394

[79] Morel O van Beekhuizen HJ Braun T Collins S Pateisky P Calda P. Performance of antenatal imaging to predict placenta accreta spectrum degree of severity. Acta Obstet Gynecol Scand (2021) 100 Suppl 1:21–8. doi: 10.1111/aogs.14112 PMC8252006 33811333

[80] Bartels HC Postle JD Downey P Brennan DJ. Placenta accreta spectrum: A review of pathology, molecular biology, and biomarkers. Dis Markers (2018) 2018:1507674. doi: 10.1155/2018/1507674 30057649 PMC6051104

[81] Li J Zhang N Zhang Y Hu X Gao G Ye Y. Human placental lactogen mRNA in maternal plasma play a role in prenatal diagnosis of abnormally invasive placenta: yes or no? Gynecol Endocrinol (2019) 35(7):631–4. doi: 10.1080/09513590.2019.1576607 30784325

[82] Choi SY Yun J Lee OJ Han HS Yeo MK Lee MA. MicroRNA expression profiles in placenta with severe preeclampsia using a PNA-based microarray. Placenta (2013) 34(9):799–804. doi: 10.1016/j.placenta.2013.06.006 23830491

[83] Gunel T Hosseini MK Gumusoglu E Kisakesen HI Benian A Aydinli K. Expression profiling of maternal plasma and placenta microRNAs in preeclamptic pregnancies by microarray technology. Placenta (2017) 52:77–85. doi: 10.1016/j.placenta.2017.02.019 28454701

[84] Hosseini MK Gunel T Gumusoglu E Benian A Aydinli K.. MicroRNA expression profiling in placenta and maternal plasma in early pregnancy loss. Mol Med Rep (2018) 17(4):4941–52. doi: 10.3892/mmr.2018.8530 PMC5865953 29393376

[85] Dillies MA Rau A Aubert J Hennequet-Antier C Jeanmougin M Servant N. A comprehensive evaluation of normalization methods for illumina high-throughput RNA sequencing data analysis. Brief Bioinform (2013) 14(6):671–83. doi: 10.1093/bib/bbs046 22988256

[86] Plotnikova O Baranova A Skoblov M. Comprehensive analysis of human microRNA-mRNA interactome. Front Genet (2019) 10:933. doi: 10.3389/fgene.2019.00933 31649721 PMC6792129

[87] Chen S Pang D Li Y Zhou J Liu Y Yang S. Serum miRNA biomarker discovery for placenta accreta spectrum. Placenta (2020) 101:215–20. doi: 10.1016/j.placenta.2020.09.068 33017714

[88] Timofeeva AV Fedorov IS Pirogova MM Vasilchenko ON Chagovets VV Ezhova LS. Clusterin and its potential regulatory microRNAs as a part of secretome for the diagnosis of abnormally invasive placenta: Accreta, increta, and percreta cases. Life (Basel) (2021) 11(4):270. doi: 10.3390/life11040270 33805203 PMC8064394

[89] Li H Ge Q Guo L Lu Z. Maternal plasma miRNAs expression in preeclamptic pregnancies. BioMed Res Int (2013) 2013:970265. doi: 10.1155/2013/970265 24195082 PMC3781840

[90] Morales Prieto DM Markert UR. MicroRNAs in pregnancy. J Reprod Immunol (2011) 88(2):106–11. doi: 10.1016/j.jri.2011.01.004 21353310

[91] Li X Shen L Shang X Liu W. Subpathway analysis based on signaling-pathway impact analysis of signaling pathway. PloS One (2015) 10(7):e0132813. doi: 10.1371/journal.pone.0132813 26207919 PMC4514860

[92] Duan L Schimmelmann M Wu Y Reisch B Faas M Kimmig R. CCN3 signaling is differently regulated in placental diseases preeclampsia and abnormally invasive placenta. Front Endocrinol (Lausanne) (2020) 11:597549. doi: 10.3389/fendo.2020.597549 33304321 PMC7701218

[93] Karmakar S Das C. Regulation of trophoblast invasion by IL-1beta and TGF-beta1. Am J Reprod Immunol (2002) 48(4):210–9. doi: 10.1034/j.1600-0897.2002.01151.x 12516631

[94] Haque S Morris JC. Transforming growth factor-β: A therapeutic target for cancer. Hum Vaccin Immunother (2017) 13(8):1741–50. doi: 10.1080/21645515.2017.1327107 PMC5557219 28575585

[95] Cheng JC Chang HM Leung PC. Transforming growth factor-beta1 inhibits trophoblast cell invasion by inducing snail-mediated down-regulation of vascular endothelial-cadherin protein. J Biol Chem (2013) 288(46):33181–92. doi: 10.1074/jbc.M113.488866 PMC3829165 24106276

[96] Shih JC Lin HH Hsiao AC Su YT Tsai S Chien CL. Unveiling the role of microRNA-7 in linking TGF-beta-Smad-mediated epithelial-mesenchymal transition with negative regulation of trophoblast invasion. FASEB J (2019) 33(5):6281–95. doi: 10.1096/fj.201801898RR 30789794

[97] El-Hussieny M Mohammed EM Zenhom NM Refaie MM Okasha AM Tawab MAE. Possible role of TGF-. Fetal Pediatr Pathol (2021) 40(3):222–32. doi: 10.1080/15513815.2020.1843574 33172328

[98] Makker A Goel MM Nigam D Mahdi AA Das V Agarwal A. Aberrant akt activation during implantation window in infertile women with intramural uterine fibroids. Reprod Sci (2018) 25(8):1243–53. doi: 10.1177/1933719117737844 29113583

[99] Tokyol C Aktepe F Hüsniye Dilek F Yilmazer M. Comparison of placental PTEN and beta1 integrin expression in early spontaneous abortion, early and late normal pregnancy. Ups J Med Sci (2008) 113(2):235–42. doi: 10.3109/2000-1967-231 18509818

[100] Xiao J Tao T Yin Y Zhao L Yang L Hu L. miR-144 may regulate the proliferation, migration and invasion of trophoblastic cells through targeting PTEN in preeclampsia. BioMed Pharmacother (2017) 94:341–53. doi: 10.1016/j.biopha.2017.07.130 28772212

[101] Lou CX Zhou XT Tian QC Xie HQ Zhang JY. Low expression of microRNA-21 inhibits trophoblast cell infiltration through targeting PTEN. Eur Rev Med Pharmacol Sci (2018) 22(19):6181–9. doi: 10.26355/eurrev_201810_16023 30338782

[102] Araki K Kawauchi K Tanaka N. IKK/NF-kappaB signaling pathway inhibits cell-cycle progression by a novel Rb-independent suppression system for E2F transcription factors. Oncogene (2008) 27(43):5696–705. doi: 10.1038/onc.2008.184 18542057

[103] Xue P Zheng M Diao Z Shen L Liu M Gong P. miR-155* mediates suppressive effect of PTEN 3'-untranslated region on AP-1/NF-kappaB pathway in HTR-8/SVneo cells. Placenta (2013) 34(8):650–6. doi: 10.1016/j.placenta.2013.04.015 23684381

[104] Xie M Fu Z Cao J Liu Y Wu J Li Q. MicroRNA-132 and microRNA-212 mediate doxorubicin resistance by down-regulating the PTEN-AKT/NF-kappaB signaling pathway in breast cancer. BioMed Pharmacother (2018) 102:286–94. doi: 10.1016/j.biopha.2018.03.088 29567542

[105] Tian Y Li H Qiu T Dai J Zhang Y Chen J. Loss of PTEN induces lung fibrosis via alveolar epithelial cell senescence depending on NF-kappaB activation. Aging Cell (2019) 18(1):e12858. doi: 10.1111/acel.12858 30548445 PMC6351835

[106] Yang H Kong W He L Zhao JJ O'Donnell JD Wang J. MicroRNA expression profiling in human ovarian cancer: miR-214 induces cell survival and cisplatin resistance by targeting PTEN. Cancer Res (2008) 68(2):425–33. doi: 10.1158/0008-5472.CAN-07-2488 18199536

[107] Kato M Putta S Wang M Yuan H Lanting L Nair I. TGF-beta activates akt kinase through a microRNA-dependent amplifying circuit targeting PTEN. Nat Cell Biol (2009) 11(7):881–9. doi: 10.1038/ncb1897 PMC2744130 19543271

