# Supplementary material for: Identification of altered miRNAs and their targets in placenta accreta
Source: Front Endocrinol (Lausanne). 2023 Mar 3;14:1021640. doi: 10.3389/fendo.2023.1021640 (PMC10022468; doi:10.3389/fendo.2023.1021640)
Supplement: Supplementary file 1 [file Table_1.docx]

Supplementary Material

# Supplementary Table 1. Placenta-associated clusters. List of miRNAs included in each cluster.

| C14MC |  |
| --- | --- |
| name | ID |
| hsa-mir-379 | ENSG00000199088 |
| hsa-mir-411 | ENSG00000199109 |
| hsa-mir-299 | ENSG00000207749 |
| hsa-mir-380 | ENSG00000198982 |
| hsa-mir-1197 | ENSG00000221745 |
| hsa-mir-323A | ENSG00000199069 |
| hsa-mir-758 | ENSG00000211582 |
| hsa-mir-329-1 | ENSG00000207761 |
| hsa-mir-329-2 | ENSG00000207762 |
| hsa-mir-494 | ENSG00000194717 |
| hsa-mir-1193 | ENSG00000221036 |
| hsa-mir-543 | ENSG00000212040 |
| hsa-mir-495 | ENSG00000207743 |
| hsa-mir-376C | ENSG00000283279 |
| hsa-mir-376A2 | ENSG00000283561 |
| hsa-mir-654 | ENSG00000207934 |
| hsa-mir-376B | ENSG00000283556 |
| hsa-mir-376A1 | ENSG00000283588 |
| hsa-mir-300 | ENSG00000215957 |
| hsa-mir-1185-1 | ENSG00000221525 |
| hsa-mir-1185-2 | ENSG00000221614 |
| hsa-mir-381 | ENSG00000199020 |
| hsa-mir-487B | ENSG00000207754 |
| hsa-mir-539 | ENSG00000202560 |
| hsa-mir-889 | ENSG00000216099 |
| hsa-mir-544A | ENSG00000207587 |
| hsa-mir-655 | ENSG00000207646 |
| hsa-mir-487A | ENSG00000207742 |
| hsa-mir-382 | ENSG00000283170 |
| hsa-mir-134 | ENSG00000207993 |
| hsa-mir-668 | ENSG00000276352 |
| hsa-mir-485 | ENSG00000208027 |
| hsa-mir-323B | ENSG00000208004 |
| hsa-mir-154 | ENSG00000207978 |
| hsa-mir-496 | ENSG00000207961 |
| hsa-mir-377 | ENSG00000199015 |
| hsa-mir-541 | ENSG00000216179 |
| hsa-mir-409 | ENSG00000199107 |
| hsa-mir-412 | ENSG00000199012 |
| hsa-mir-369 | ENSG00000199025 |
| hsa-mir-410 | ENSG00000199092 |
| hsa-mir-656 | ENSG00000207959 |
|  |  |
| C19MC |  |
| name | ID |
| hsa-mir-512-1 | ENSG00000207645 |
| hsa-mir-512-2 | ENSG00000207644 |
| hsa-mir-1323 | ENSG00000221017 |
| hsa-mir-498 | ENSG00000207869 |
| hsa-mir-520E | ENSG00000207599 |
| hsa-mir-515-1 | ENSG00000207616 |
| hsa-mir-519E | ENSG00000207810 |
| hsa-mir-520F | ENSG00000283540 |
| hsa-mir-515-2 | ENSG00000207615 |
| hsa-mir-519C | ENSG00000207788 |
| hsa-mir-1283-1 | ENSG00000221421 |
| hsa-mir-520A | ENSG00000207594 |
| hsa-mir-526B | ENSG00000207580 |
| hsa-mir-519B | ENSG00000207825 |
| hsa-mir-525 | ENSG00000207711 |
| hsa-mir-523 | ENSG00000283455 |
| hsa-mir-518F | ENSG00000207706 |
| hsa-mir-520B | ENSG00000207722 |
| hsa-mir-518B | ENSG00000207862 |
| hsa-mir-526A1 | ENSG00000207629 |
| hsa-mir-520C | ENSG00000207738 |
| hsa-mir-518C | ENSG00000283490 |
| hsa-mir-524 | ENSG00000283289 |
| hsa-mir-517A | ENSG00000207734 |
| hsa-mir-519D | ENSG00000207981 |
| hsa-mir-521-2 | ENSG00000207549 |
| hsa-mir-520D | ENSG00000207735 |
| hsa-mir-517B | ENSG00000207837 |
| hsa-mir-520G | ENSG00000207799 |
| hsa-mir-516B2 | ENSG00000207925 |
| hsa-mir-526A2 | ENSG00000211532 |
| hsa-mir-518E | ENSG00000207987 |
| hsa-mir-518A1 | ENSG00000207803 |
| hsa-mir-518D | ENSG00000283330 |
| hsa-mir-516B1 | ENSG00000207946 |
| hsa-mir-518A2 | ENSG00000207699 |
| hsa-mir-517C | ENSG00000207838 |
| hsa-mir-520H | ENSG00000207861 |
| hsa-mir-521-1 | ENSG00000207634 |
| hsa-mir-522 | ENSG00000283685 |
| hsa-mir-519A1 | ENSG00000207992 |
| hsa-mir-527 | ENSG00000207979 |
| hsa-mir-516A1 | ENSG00000207767 |
| hsa-mir-1283-2 | ENSG00000221548 |
| hsa-mir-516A2 | ENSG00000207620 |
| hsa-mir-519A2 | ENSG00000284362 |
|  |  |
| mir-371 cluster |  |
| name | ID |
| hsa-mir-371A | ENSG00000199031 |
| hsa-mir-371B | ENSG00000284568 |
| hsa-mir-372 | ENSG00000199095 |
| hsa-mir-373 | ENSG00000199143 |
|  |  |
| mir-17/92 cluster |  |
| name | ID |
| hsa-mir-17 | ENSG00000284536 |
| hsa-mir-18A | ENSG00000283815 |
| hsa-mir-19A | ENSG00000284204 |
| hsa-mir-20A | ENSG00000283762 |
| hsa-mir-19B1 | ENSG00000284375 |
| hsa-mir-92A1 | ENSG00000283705 |
|  |  |
| miR-106a/363 cluster |  |
| name | ID |
| hsa-mir-106A | ENSG00000284157 |
| hsa-mir-18B | ENSG00000283931 |
| hsa-mir-20B | ENSG00000284043 |
| hsa-mir-19B2 | ENSG00000284107 |
| hsa-mir-92A2 | ENSG00000284538 |
| hsa-mir-363 | ENSG00000284499 |
|  |  |
| mir-106b cluster |  |
| name | ID |
| hsa-mir-106B | ENSG00000208036 |
| hsa-mir-93 | ENSG00000207757 |
| hsa-mir-25 | ENSG00000207547 |
